# Physical behavior of PEDOT polymer electrode during magnetic resonance imaging and long-term test in the climate chamber

**DOI:** 10.1038/s41598-023-33180-5

**Published:** 2023-04-10

**Authors:** Nora Vanessa de Camp, Jürgen Bergeler, Frank Seifert

**Affiliations:** 1 Petesys UG Limited, Mühlenfließ, Germany; 2 Institute for Biology, Behavioral Physiology, Humboldt University Berlin, Berlin, Germany; 3 Physikalisch-Technische Bundesanstalt (PTB), Brunswick, Berlin, Germany

**Keywords:** Biological techniques, Neuroscience, Materials for devices, Magnetic resonance imaging, Neonatology, Preterm birth

## Abstract

The PEDOT polymer electrode is a metal-free electrode, consisting of an acrylate (dental composite) and the conductive polymer poly(3,4-ethylenedioxythiophene) polystyrene sulfonate (PEDOT:PSS). The electrode is applied as gel onto the skin and cured with blue light for 10–20 s in order to achieve a conductive bond to the skin. The electrodes are used in combination with polymer cables consisting of a textile backbone and PEDOT:PSS. To test this new electrode and cable type under different conditions we designed two stress-tests: highly sensitive temperature recordings within a head phantom during Magnetic Resonance Imaging (MRI) and long-term stability inside a climate chamber with high humidity. To study the physical behavior inside the strong magnetic field (3 Tesla), the PEDOT polymer electrode was attached to an agarose head-phantom inside a magnetic resonance tomograph during an image sequence. MRI-safe temperature sensors were placed nearby in order to measure possible heating effects. In comparison to a metal cable, nearly no rise in temperature could be observed if the electrode was used in combination with a conductive textile cable. Furthermore, the electrode showed stable impedance values inside a climate chamber for 4 consecutive days. These results pave the way for testing the PEDOT polymer electrode as biosignal recording electrode during MRI, especially for cardio MRI and Electroencephalography in combination with functional MRI (EEG–fMRI).

## Introduction

The PEDOT polymer electrode^1^ is a metal free biosignal electrode, especially for EEG recordings. The electrode consists of an acrylate component, as for example dental cement and PEDOT:PSS, a conductive polymer. The electrode has a gel-like texture until it is cured with blue light for 10–20 s. The resulting electrode is self adhesive and conductive. Due to its change of aggregate state, it can be applied underneath hair without shaving. The texture is like dry mud. The material can be removed pain-free by applying pressure from two sides, for example crumbling the electrode between two fingers.

Since the electrode can also be applied on very small spaces and has a thickness of only 1 or 2 mm, it is especially well suited for small application areas, as for example children, neonates or small animals.

The two components of the electrode are polymers with frequent use:

PEDOT:PSS is a well known conductive polymer which is used in the solar industry^2^ but also for implantable electrodes^3^. It is often used to produce flexible electrodes, conductive coatings or conductive inks^4–6^.

Acrylates are widely used as ingredient of dental composites^7^. Here, we used the dental composite tetric evo flow (Ivoclar, Vivadent).

The PEDOT polymer electrode is currently the only metal-free biosignal recording electrode which is, self-adhesive and changes aggregate state within a few seconds. The tight bond to the skin makes EEG recordings during movement possible^1^. In addition, the electrode is flat and therefore does not lead to pressure points, a prerequisite for EEG recordings in the neonatal intensive care unit or in sleep laboratories. The idea is to design an electrode which is nearly invisible, small, enduring and stable under several conditions. Here, we tested two common stress-conditions for electrodes: the heating behavior in the magnetic resonance tomograph and the long term stability regarding impedance as well as resistance inside a climate chamber with high temperature and humidity. In combination with the electrode, we use a polymer cable in the form of a textile impregnated with PEDOT:PSS. Such impregnated textiles are already the subject of research (Review: ^8^).

MRI became one of the most powerful medical imaging solutions during the past decades (Review:^9^). It gives precise images of inner organs in a non-invasive way. The magnetic fields applied in MRI do not cause damage to tissues and organs but they can be extremely destructive when medical implants or other devices with metal parts are present, as for example hip implants or cardiac pacemakers. Therefore, strict safety guidelines must be followed, when working with MRI^10^. The Review on Magnetic Resonance Safety^11^ presents the advantages of the technique, in particular the high resolution of soft tissue images but also risks, such as force on ferromagnetic objects, heating of tissues, peripheral nerve stimulation, hearing damage, implants, contrast agents, and imaging during pregnancy.

Various materials for MRI-safe electrodes have been developed and tested in the past. For example implantable Electrodes on the basis of carbon nanotubes^12^. MRI safety was also reported for implantable platinum electrode arrays in a clinical review^13^.

In the case of non-invasive EEG electrodes, various materials have also been evaluated as safe: gold-plated copper electrodes with silver wires^14^ large-area epidermal electronics with the conductive materials chromium and silver^15^ and head caps^16^. Notably, few non-invasive systems are tested under the applicable ASTM standards for MRI safety^17^.

We expect a certain heating of the electrode during MRI examination, especially in case of a loop with copper wires, due to well described radiofrequency (RF) interactions with loops^18^. Regarding long-term stability in the climate chamber, we expect better results in case of the copper wire, because it is insulated, in contrast to the polymer cables. A reasonable scenario is also the fragmentation of the PEDOT polymer electrode and a resulting loss of contact to the cable.

Our results show, that the electrode is stable under conditions of high humidity and temperature and that heating of the electrode in the MRI scanner is below 1 °C.

## Material and methods

### Electrode

1:1 Mixture of Clevios SV3 (Heraeus, Leverkusen, Germany) and Tetric Evo Flow (Ivoclar Vivadent, Germany).

### Magnetic resonance tomograph

**Figure 1:**
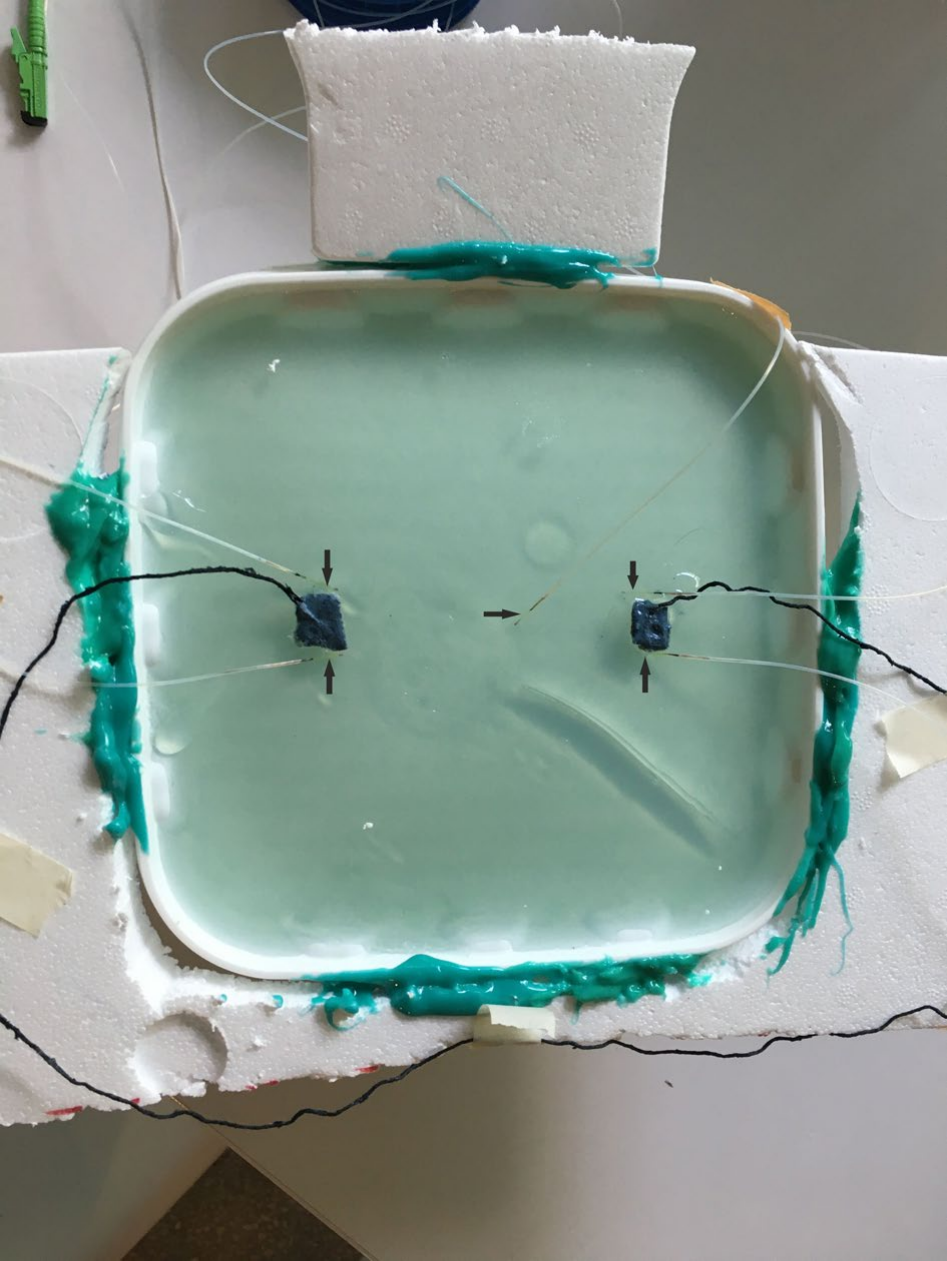
Head phantom for MRI with PEDOT polymer electrode and conductive textile wire. Top view on the agarose filled plastic bottle. The lid is also filled with agarose and two small pieces of gauze are embedded in the agarose as anchor points for the electrode. The polymer electrode and the cable are attached to these two points (black). The cable connecting the two electrodes is fixed as a loop on a styrofoam frame to force possible heating effects. Next to the polymer electrode, 2 temperature sensors are embedded in the upper layer of the agarose (white fiber strands). Another temperature sensor is placed as a reference in the middle of the lid, between the electrodes. The embedded tips of the fibre optic temperature sensors are marked with black arrows.

**Figure 2:**
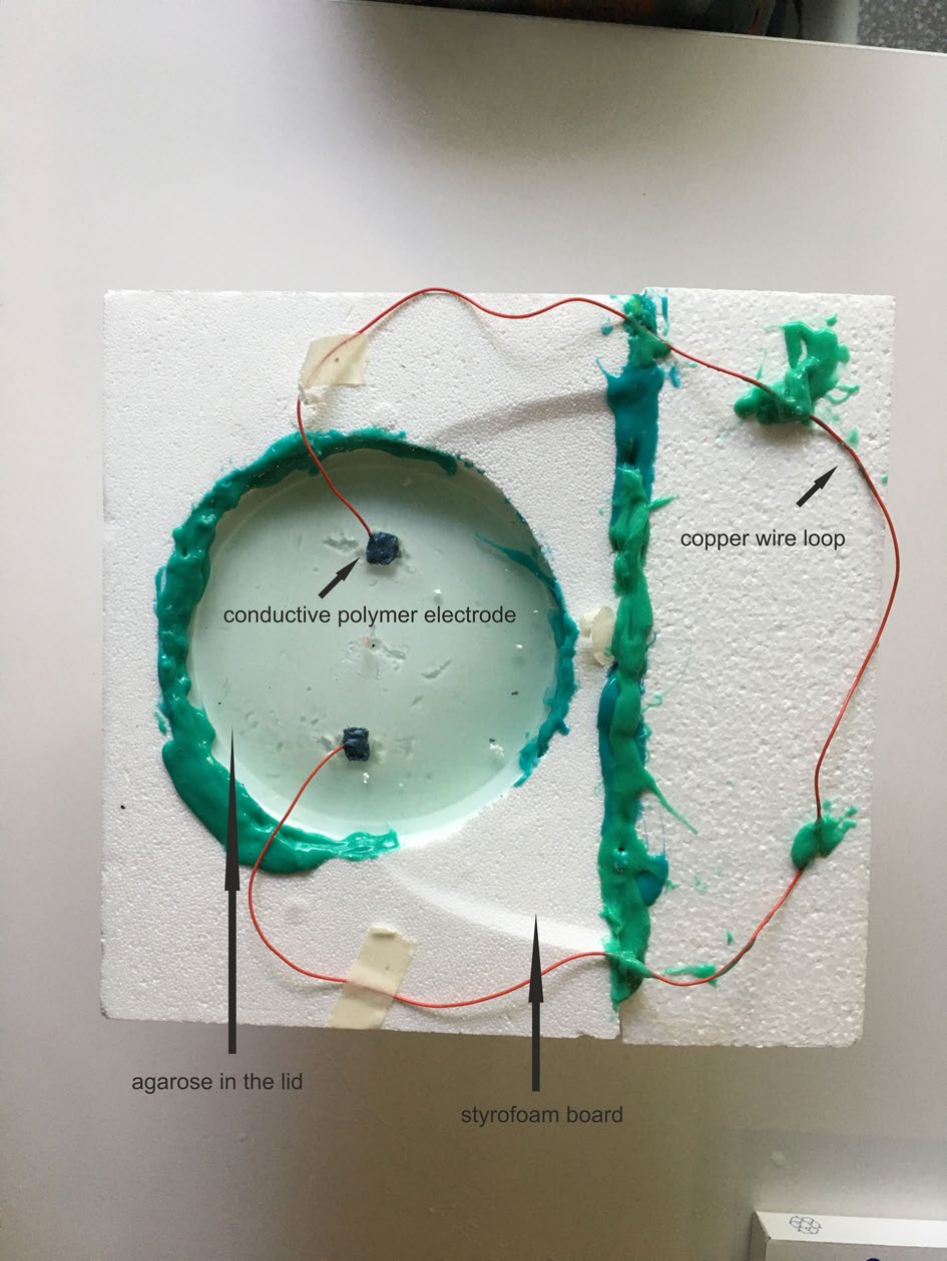
Head phantom for MRI with PEDOT polymer electrode (black) and metal wire. Similar setup as in Fig. 1 but with insulated metal leads (red). The styrofoam board is fixed with silicone (body double fast, Smooth on Inc., USA).

**Figure 3:**
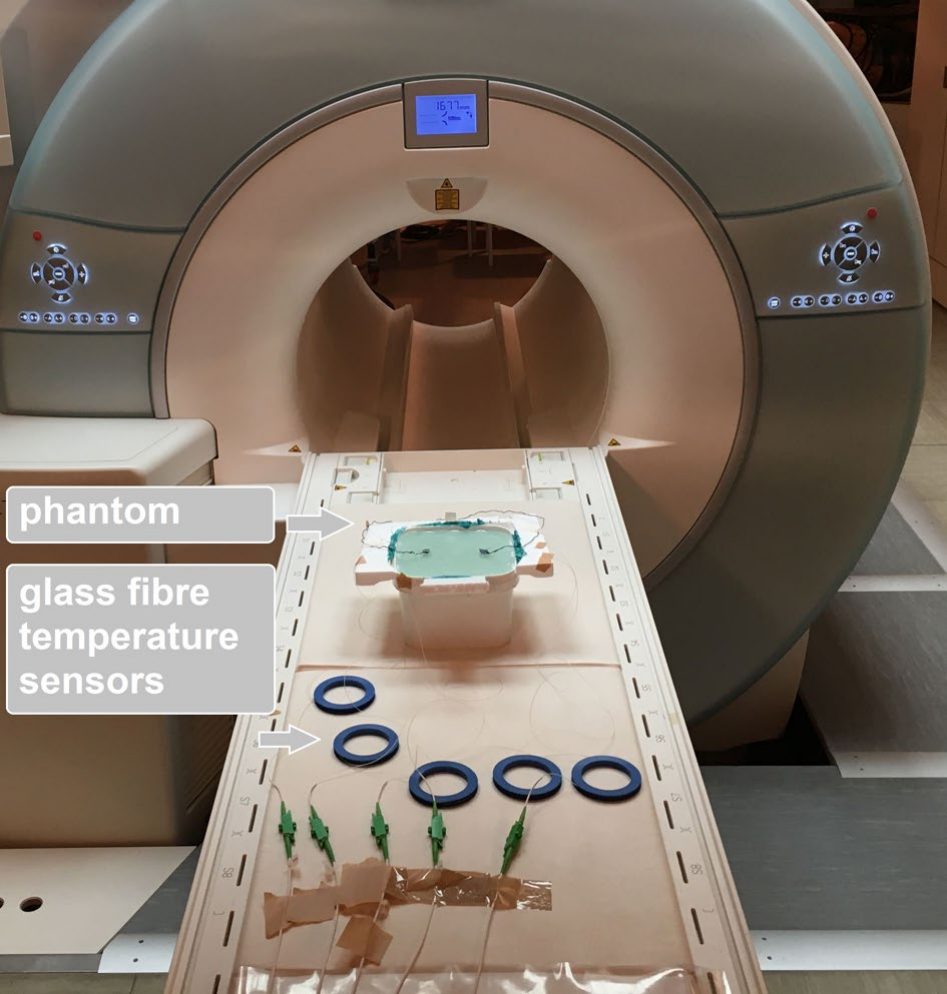
Setup with MRI. In the foreground, the temperature sensors are attached to the table, behind them is the head phantom with the electrodes and cables.

A 3 Tesla MRI scanner (Siemens Magnetom Verio) was used to test the electrode. A 3 L plastic bottle was filled completely and bubble-free with agarose (1%agarose, 0.67 g/L CuSO4 and 1.33 g/L NaCl). The plastic bottle was closed and the cap was filled with the same agarose solution (2 cm thick layer on the lid). An established and very well described cuboid agarose phantom served as a template for this phantom^19^. Two small pieces of mull tissue were immersed in the agarose before gelation in order to have a fixation point for the polymer electrodes. The PEDOT:PSS polymer electrodes (in the following only polymer electrode) were administered on top of the agarose soaked mull together with a conductive textile wire. Application of blue light for 20 s cured the electrode and fixed the textile wire inside the polymer electrode (round shape with 1 cm diameter, maximal thickness of 2 mm). A dental LED curing light (Valo Cordless, Ultradent, Utah, USA) with a spectrum of 395–480 nm was used. The PEDOT:PSS textile “wire” (in the following conductive textile wire) consists of bag sewing yarn (synthetic mixed fibre) soaked in PEDOT:PSS (Clevios FET, Heraeus, Germany, 6 h at room temperature) and dried 3 times at room temperature (22 °C) for 8 h, respectively. Repeated dipping and drying served to accumulate conductive polymer on the yarn and thus increased its conductivity. The weight of the textile cable doubles after this procedure, so that an approximately 50% content of Clevios FET (PEDOT:PSS formulation) can be inferred in the conductive textile wire. The conductive textile wire was placed as coil between two PEDOT polymer electrodes on top of a styrofoam board around the head phantom (Fig. 1). The styrofoam is fixed with green silicone (body double fast, Smooth On Inc., Pennsylvania, USA). A similar setup was constructed, but the conductive textile wire was replaced by a copper wire (insulated copper lead, Fig. 2). MRI-safe glass fiber temperature sensors (FBG/FBG-TEMP-XXS with CANFDX/L-FBG-T8, imc Test & Measurement GmbH Berlin, Germany) were placed as close as possible to each electrode inside the agarose. The temperature was measured during an imaging session in the magnet resonance tomograph for each condition (metal wire/conductive textile wire). The setup directly before the measurement is shown in Fig. 3. In the foreground, the temperature sensors are visible, whose measuring probes are embedded in the agarose. Behind this is the agarose box as a head phantom with the electrodes and cables. During the temperature measurement, the entire table is moved into the tomograph and subjected to an image sequence. The length of the wires is about 80 cm, diameter of about 2 mm, the electrode size is about 1.2 cm diameter. The resistance of the copper cables of 80 cm length is 1 Ω, the resistance of the PEDOT polymer cables of 80 cm length is 10 kΩ. Resistance was measured by connecting both ends of the conductive textile wire to a digital multimeter (Owon B35T, Pollin Electronics GmbH, Pförring, Germany).

The MR images of the phantom X (Fig. 12) were acquired using an 3D MPRAGE (Magnetization Prepared RApid Gradient Echo) sequence with the following imaging parameters:repetition time TR = 1900 ms, preparation time TI = 900 ms, echo time TE = 2.22 ms, imaging matrix 256 × 216 × 208, dx = 0.98 mm, dy = 1.02 mm, dz = 1,00 mm. The total scan time was 359 s.

A single loop standard receive surface coil was used to enhance the signal-to-noise of the MR images. This coil was positioned approximately 10 cm above the phantom. The SAR (specific absorption rate) was so low during this sequence that no significant heating was observed.

A multi slice TSE (Turbo Spin Echo) sequence was used to intentionally heat the phantom by a measurable amount. To this end, the surface coil was removed without changing anything in the remaining configuration. The MR images acquired with this sequence were not used further, because the body coil used has a very poor receive sensitivity. The sequence parameters were as follows:

TR = 182 ms, TE = 9.6 ms, turbo factor: 18, averages: 4, total scan time, 446 s, number of slices: 25, slice thickness: 8 mm, slice distance: 10.4 mm, field of view: 220 mm × 220 mm.

The fictive body weight was 75 kg and the scanner reported for this sequence a whole body SAR of 1.4 W/kg (averaged over 6 min). This was the maximum possible value in this configuration, since the scanner can use other limits internally (e.g. partial body SAR, see IEC 60,601-2-33), but this is not directly disclosed by the scanner software.

### Simulation

A worst case scenario has been simulated (XFDTD 6.4, Remcom Inc.) using a proper model of the RF body coil of the scanner. A perfectly conducting (PEC) wire (80 cm length and 4 mm^2^ cross section) and just such electrodes (12 mm diameter and 4 mm thickness) were assumed. The phantom was simulated as a cube with 20 cm edge length. The wire loop was also set to 20 cm × 20 cm. It is assumed that this wire loop has a resistance of 1 kOhm, which means a conductivity of 200 S/m (sigma200). In another scenario, a value of 20 S/m (Sigma20) is assumed, which corresponds to exactly 10 kΩ for the wire loop. The formula is: ${\upkappa } = \frac{{\text{l}}}{{{\text{AR}}}}$, with l = length of the electrical conductor, A = diameter of the electrical conductor and R = resistance, with the unit $\left[ {\frac{1}{{{\Omega m}}}} \right]$ = [S/m] (Siemens per meter).

### Climate chamber

**Figure 4:**
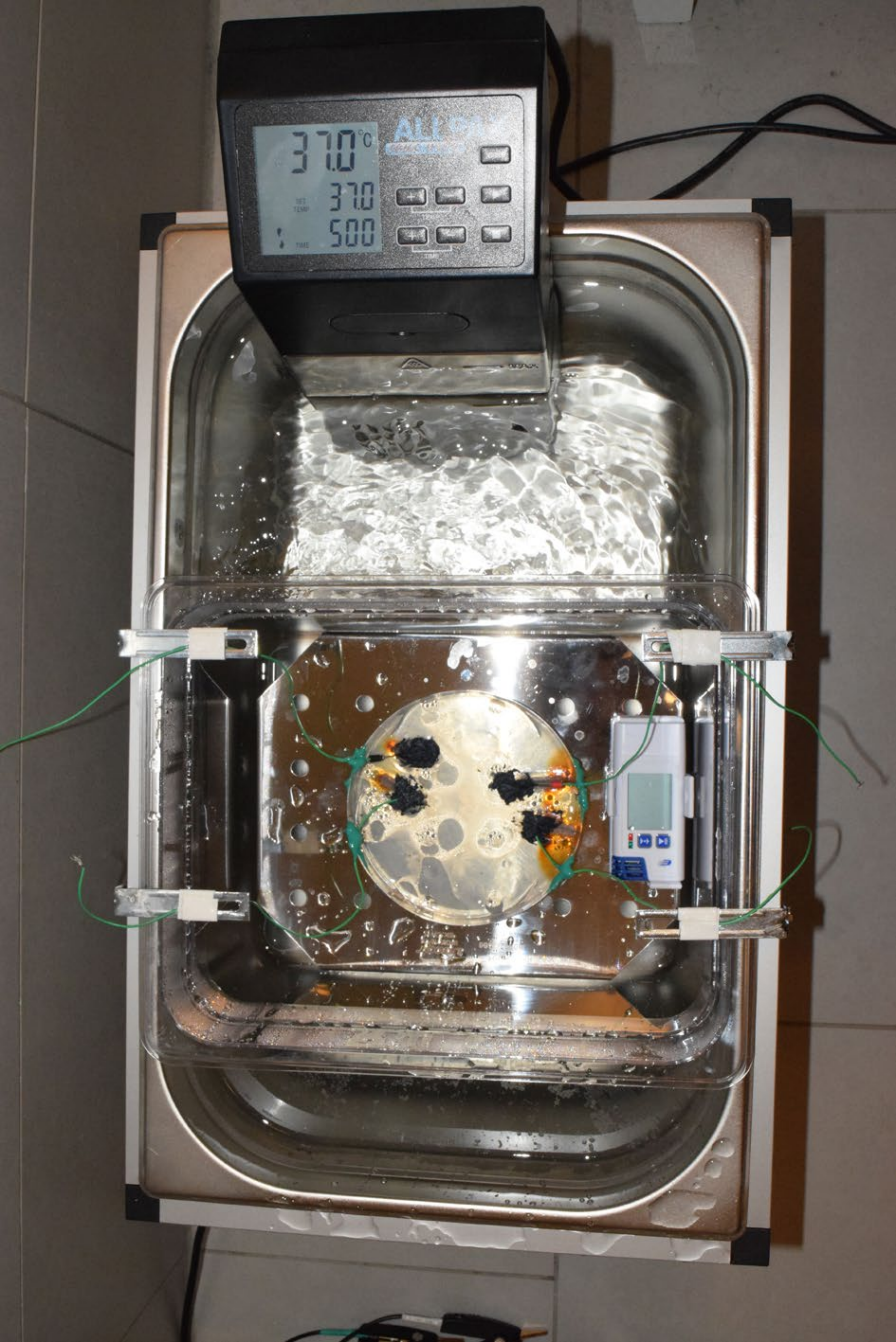
The climate chamber setup. The outer water basin is hold on a constant temperature. The inner basin is filled with water underneath a perforated metal-plate. A petri dish with the PEDOT polymer electrodes is placed above the metal plate. A short piece of pipe cleaner was inserted inside the agarose for better adhesion of the electrode (the pipe cleaners inner wire leads to orange corrosion spots on the agarose). Recording wire endings are outside the chamber (green leads). The data logger is in direct vicinity to the petri dish.

**Figure 5:**
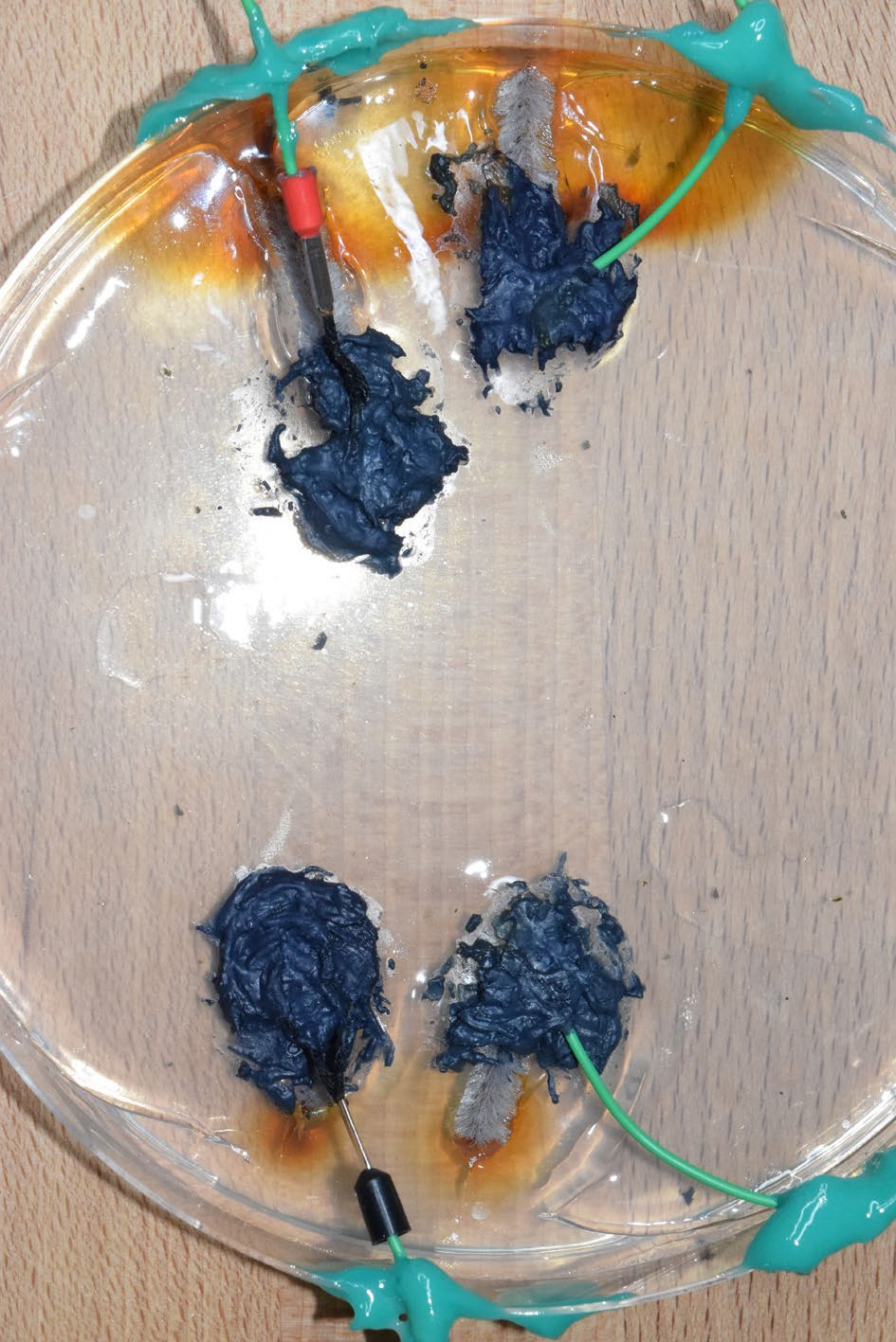
PEDOT Polymer electrode on agarose plate. Right: green metal leads directly in the electrode, Left: conductive textile interface between electrode and metal wire. Electrode diameter: 2 cm.

We used an insulated stainless-steel basin of 50 cm × 30 cm (depth of 25 cm), filled with water (Fig. 4). An immersion heating system kept the temperature of the water constantly on 37° and the relative air humidity between 84 and 92% (full content of the original data logger in the supplementary material). Agitation of the water ensured a homogenous heating. A smaller plastic basin (30 cm × 23 cm) was submerged into the outer water bath. A perforated metal floor plate allowed for an additional water deposit in the small basin, to reach high levels of humidity in the small chamber. On top of this floor plate in the small basin, a Petri dish with isotonic agarose solution (0.9 g NaCl per 100 ml) was placed next to a small data logger. The data logger (dostmann-electronic.de, LOG 220 5005-0220) recorded relative air humidity and temperature with an interval of one data point per 5 min. At four places at the edge of the agarose plate, small pieces of pipe cleaners were immersed into agarose (Fig. 5, Rust stains of the pipe cleaner in the agarose). The pipe cleaners simulated hair, because the PEDOT polymer electrodes stick better to hairy surfaces than to very smooth surfaces, like agarose. In two cases, we fixed the conductive textile cable inside the PEDOT polymer electrode. This small piece of conductive textile wire (2 cm) was then crimped to a normal wire and the endings were transferred to outside the chamber in order to measure impedance and DC resistance. DC resistance was measured with an Ohmmeter (Owon B35T, Pollin Electronics GmbH, Pförring, Germany), Impedance was measured with an Impedance measurement device (Gwinstek, LCR-6100, Precision LCR meter, 10 Hz–100 kHz). The other two PEDOT polymer electrodes were measured with the one end of the metal cable, without the textile interface. The measurement was taken for 4 consecutive days.

Data analysis took place with the internal program of the data logger as well as Matlab (2021, Mathworks). The Polymer electrodes were applied as a round shape with 2 cm diameter and maximal thickness of 2 mm.

## Results

### Climate chamber

**Figure 6:**
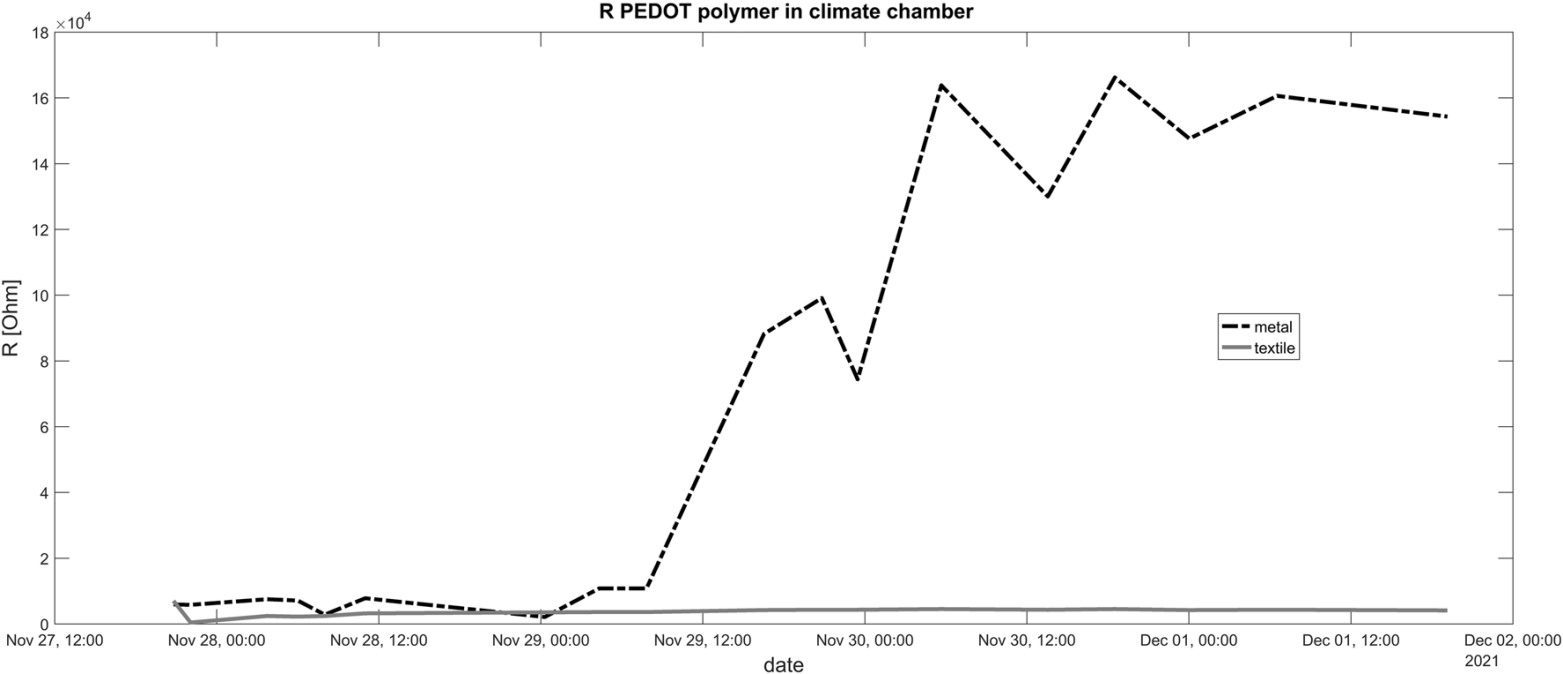
Resistance in the climate chamber for a period of several consecutive days. Contrary to expectations, the resistance of the insulated copper wires increases more than that of the polymer cables. The resistance of the polymer cables remains below 1 kΩ over the entire period. The resistance of the copper cables (in conjunction with polymer electrodes) increases to up to 160 kΩ from the 2nd day. Both cable types are connected with a polymer electrode.

**Figure 7:**
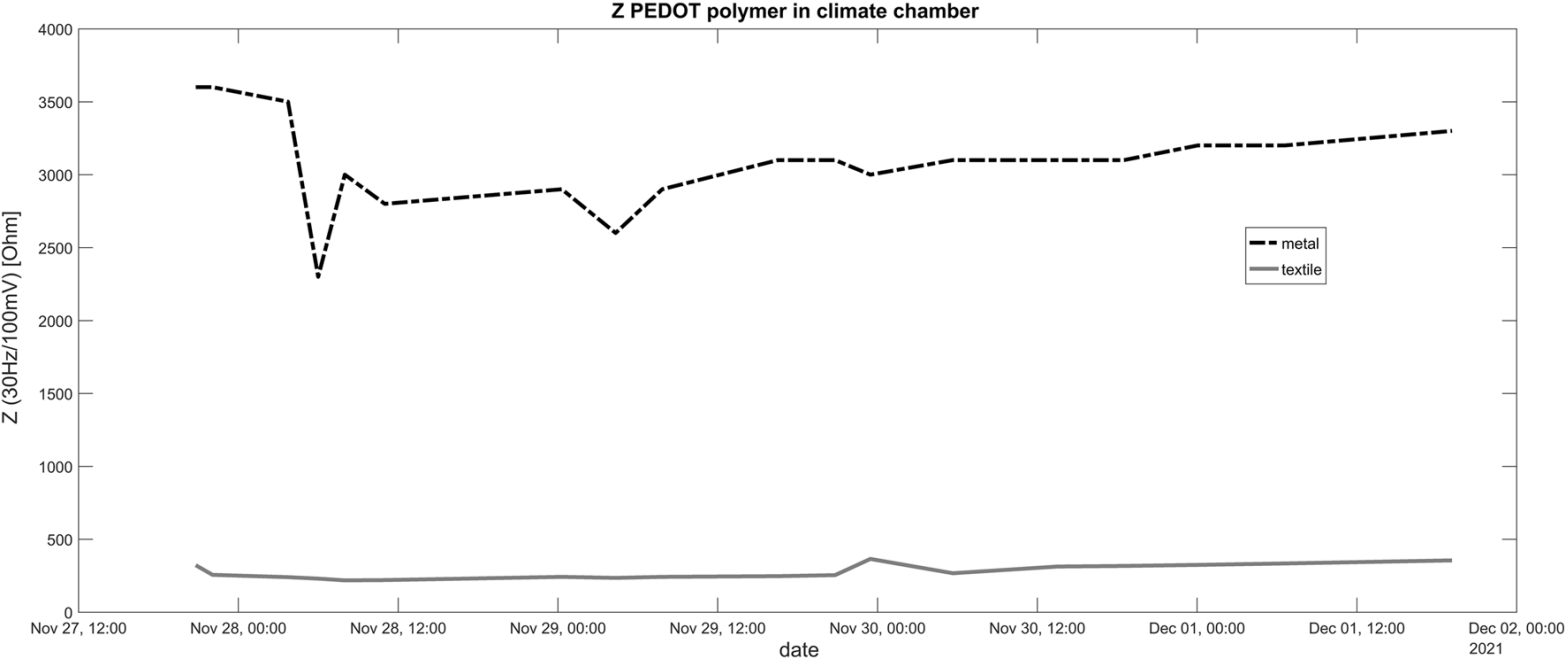
Impedance in the climate chamber for a period of several consecutive days. Similar to the results for resistance R, the impedance of the insulated copper wires increases more than that of the polymer cables. The impedance of the polymer cables remains below 1 kΩ over the entire period. The impedance of the copper cables (in conjunction with polymer electrodes) increases to up to 3.5 kΩ from the beginning on. Both cable types are connected with a polymer electrode.

Other than expected, the high air humidity had no adverse effect on the conductance or stability of the PEDOT polymer electrode. DC resistance was rising after a few days with the pure metal wire in contrast to the conductive textile wire part. The fluctuations of the DC resistance in the case of the metal cable over the measurement period are also striking. (Fig. 6). The impedance Z is stable but also lower for the conductive textile wire (Fig. 7). The data from the data logger show relatively high air humidity around 90% and constant temperature around 36 °C (original data logger content in the supplementary data).

### MRI simulation

**Figure 8:**
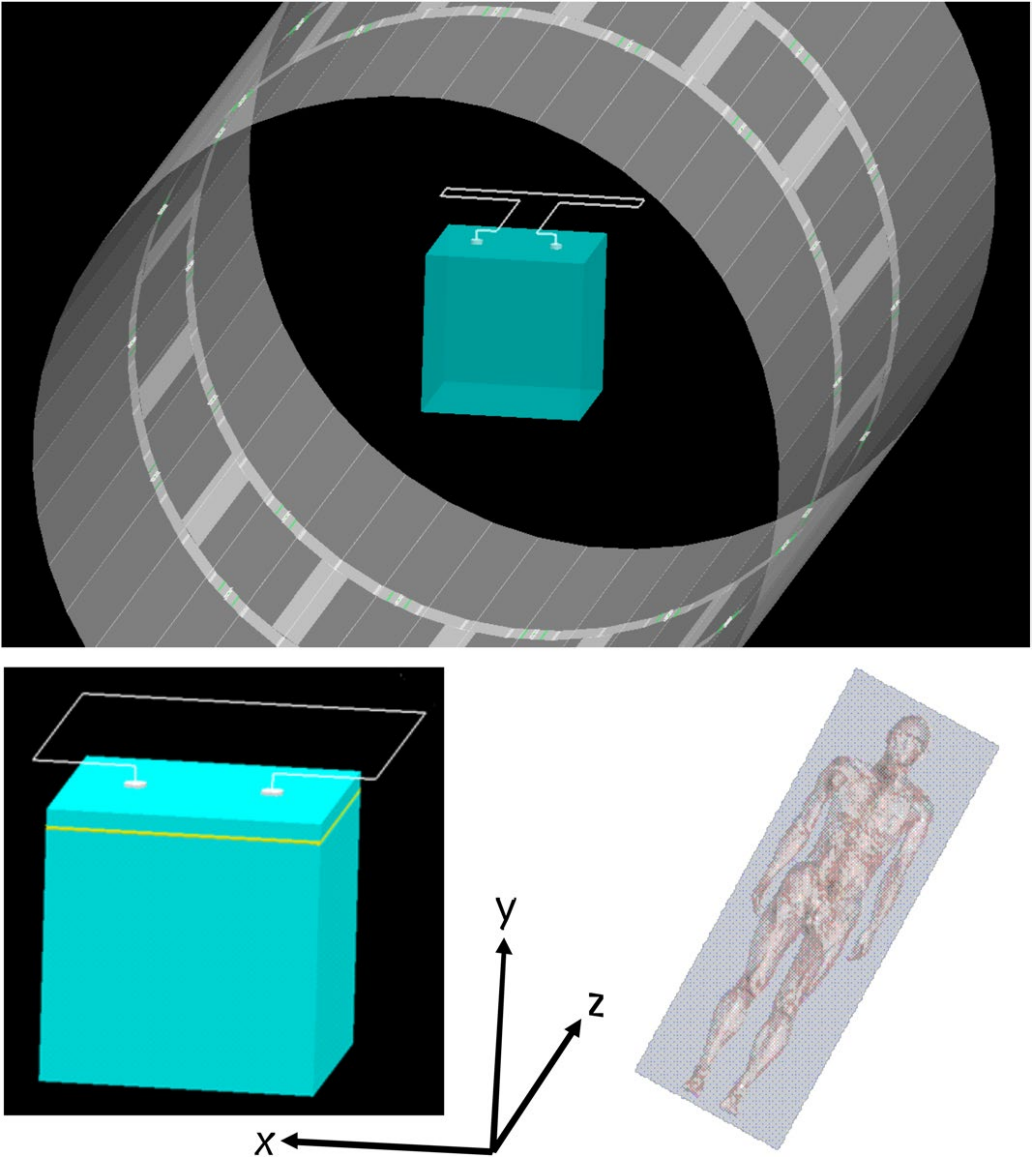
Simulation of the head phantom in MRI with leads. Small area and large area version with insulation layer (yellow). The coordinate system is intended to illustrate how the phantom is oriented in the scanner. The z direction represents the head-foot axis.

**Figure 9:**
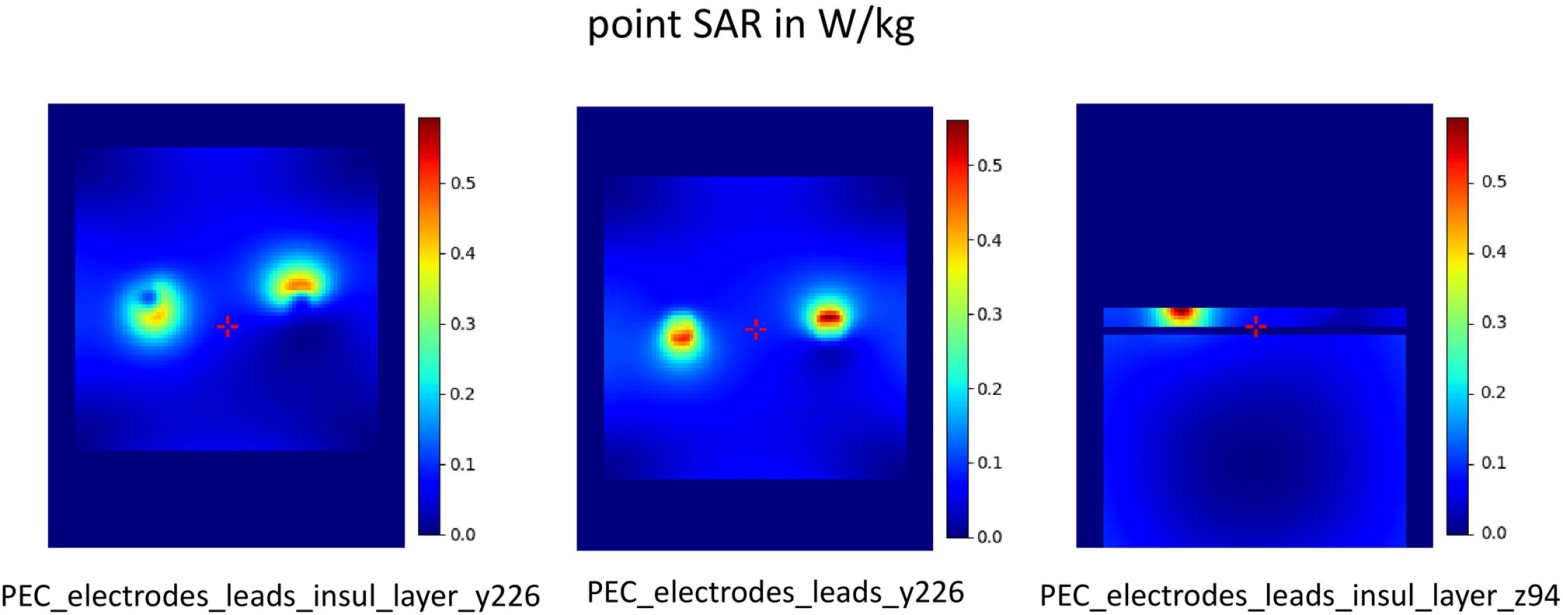
Simulation of SAR values, effect of insulation layer. The insulation layer blocks SAR values in the z-plane (right). The insulation layer weakens SAR values of a perfectly conducting electrode (left with insulation layer, middle without insulation layer).

**Figure 10:**
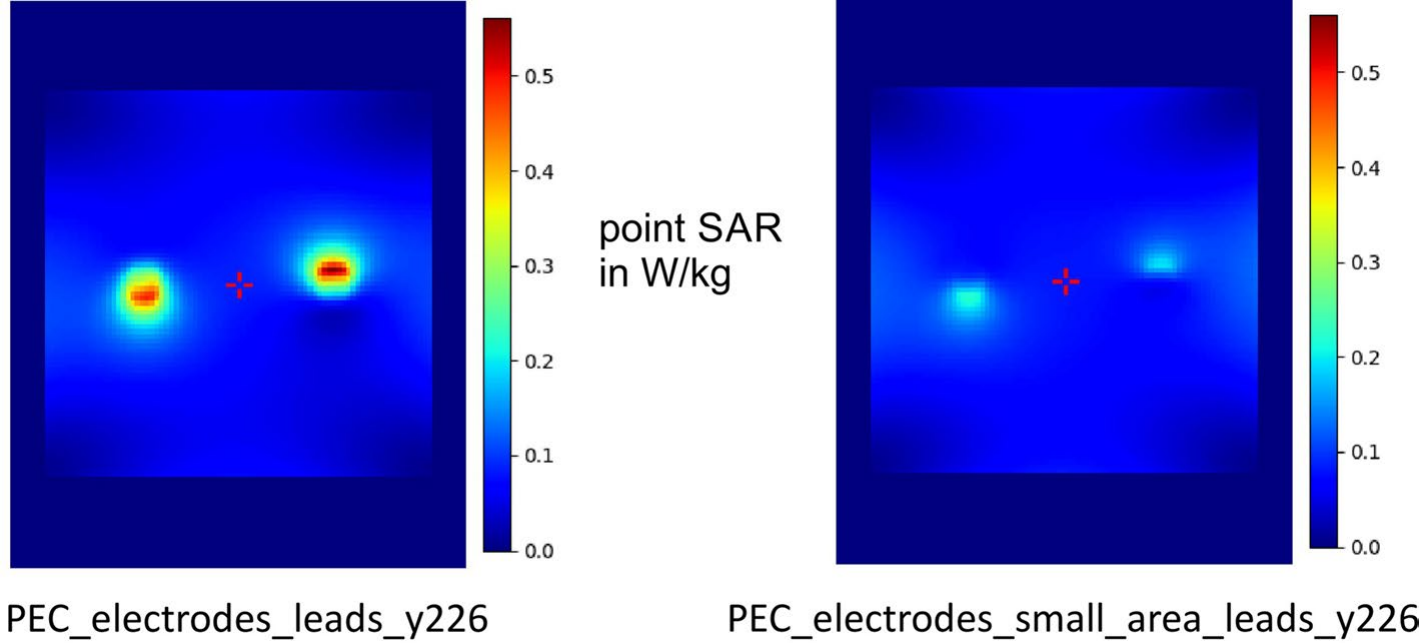
Simulation of SAR values for small area. If the leads of perfectly conducting electrodes span a small area, the SAR values are weaker. This simulation shows that the model makes correct assumptions.

**Figure 11:**
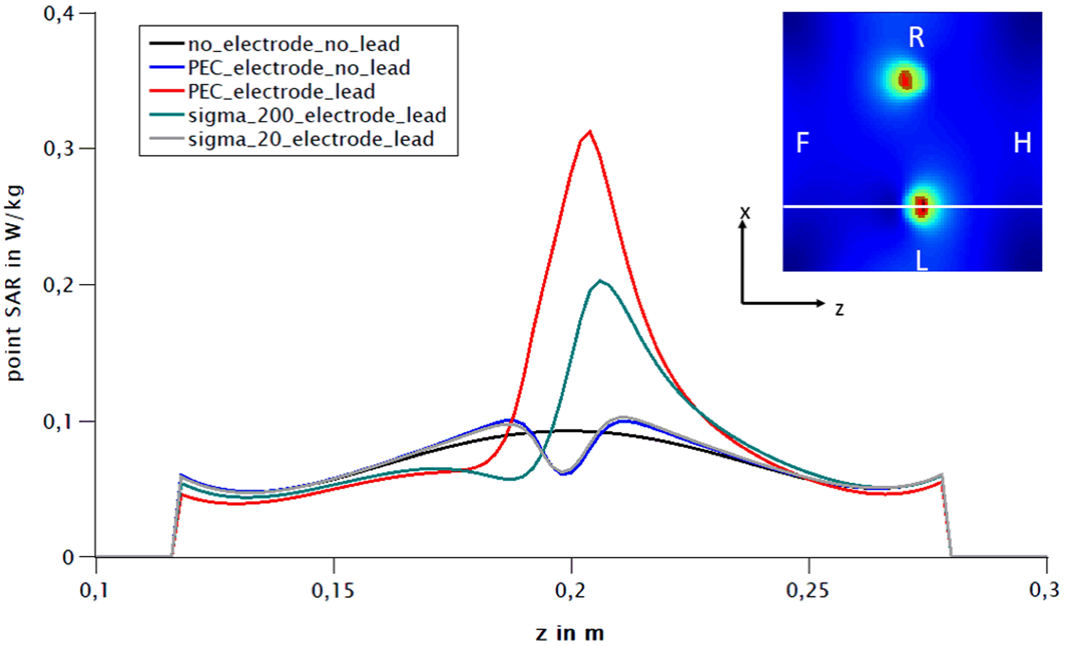
Simulation of different electrode-lead combinations and resulting (point) SAR values during MRI at B_1rms_ of 0.86 µT (see ^10^). SAR values along the z-plane as expected, a lead loop increases the SAR value. The increase of SAR is in this case proportional to the conductivity (perfect electric conductor [PEC] > Sigma 200 > Sigma20). Without wire loop, none of the electrodes shows increased SAR values. The small image on the top right shows a simulation as a heat map. The section plane for the line diagram is drawn in white. F stands for foot, H for head, R for right and L for left.

Two different basic scenarios were simulated: a small-area version, where the cables enclose a small area, and a version with a larger enclosed area and a thin insulation layer, similar to a skull and also the head phantom (Fig. 8). Without cables, the specific absorption rate (SAR) for PEC (perfect electric conductor) electrodes with an insulating layer in the phantom decrease extremely (Fig. 9). Low SAR values are also observed for PEC electrodes with cables and a small enclosed loop area (small area version, Fig. 10). PEC electrodes without any cables and the control configuration without any cables and electrodes, result in very tiny SAR values in comparison with PEC electrodes and PEC cables (Fig. 11). If the conductivity drops to 200 S/m, there are average SAR values of about 0.3 W/kg, in contrast to over 0.5 W/kg for perfect electrodes and cables. At even lower conductivity values of 20 S/m, no significant SAR value is detectable in the simulation (Fig. 11).

### Results MRI

**Figure 12:**
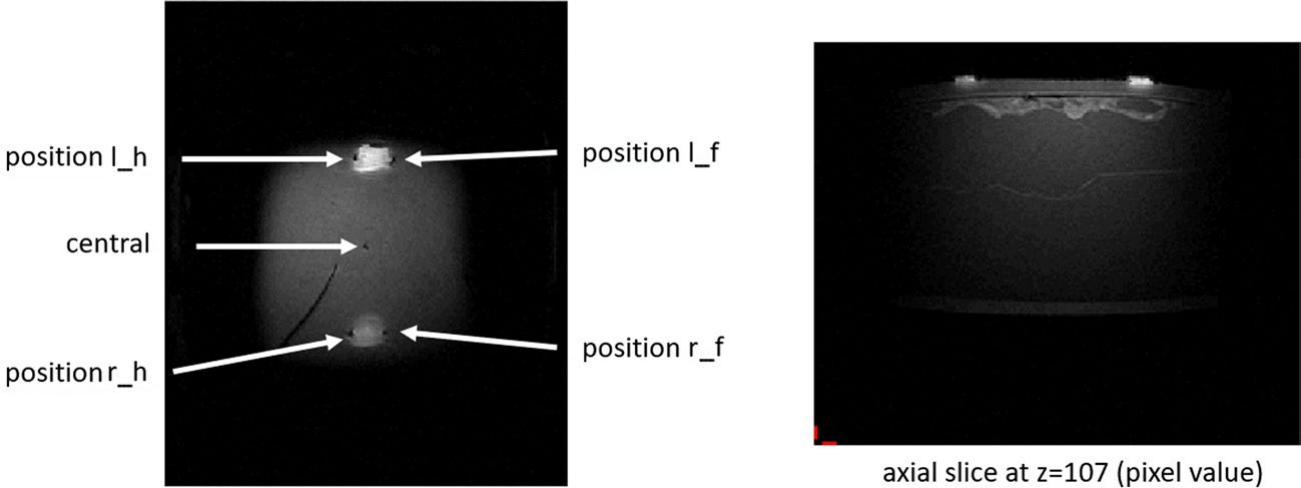
MRI image of head phantom with polymer electrodes. In the top view on the left, the temperature sensors are marked with arrows. position l_h means back left, position r_f means front right. The central temperature sensor can also be seen as a small black dot. An axial slice can be seen on the right. The interruption of the structure by the lid and slight unevenness, presumably due to air pockets in the agarose under the lid can be seen. The polymer electrodes have no significant effect on the image quality in the MRI.

**Figure 13:**
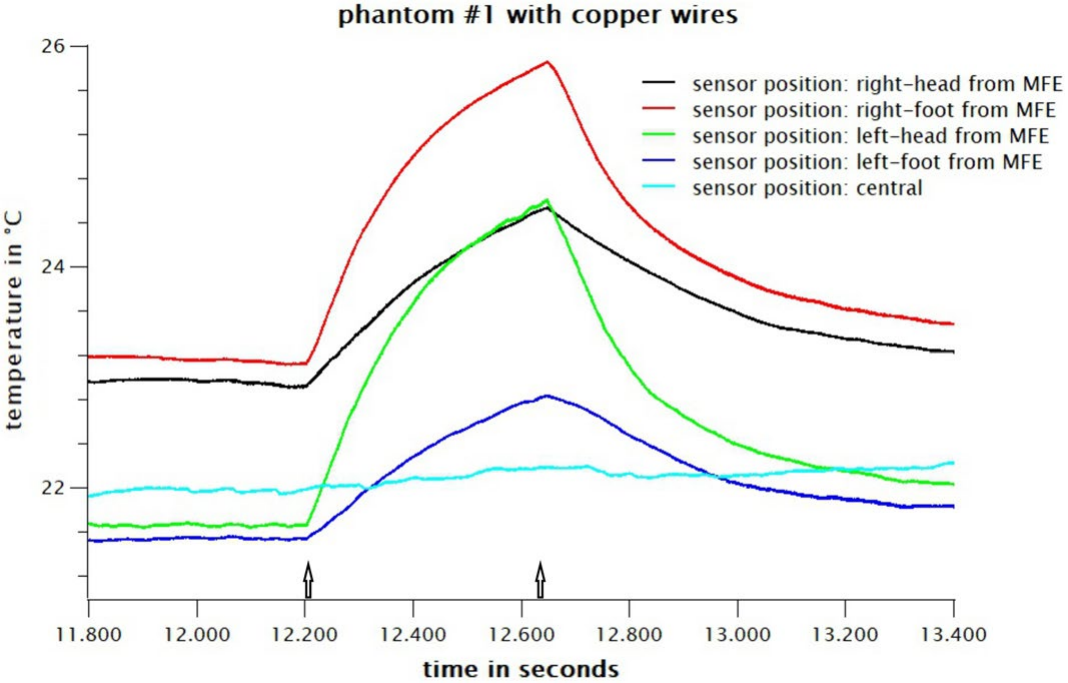
Heating effect of MRI on a polymer electrode with copper lead loop. The central temperature sensors serve as control. All other temperature sensors show heating effects up to 3 K. Only the temperature changes during the heating time are interesting, i.e. while the measurement sequence is running and high frequency is applied, i.e. between approx. 12220 s and 12660 s. The temperature sensors all have different offsets. For a better representation (otherwise all curves lie on top of each other) we have not corrected them. The beginning and the end of the respective measuring sequence are marked with an arrow.

**Figure 14:**
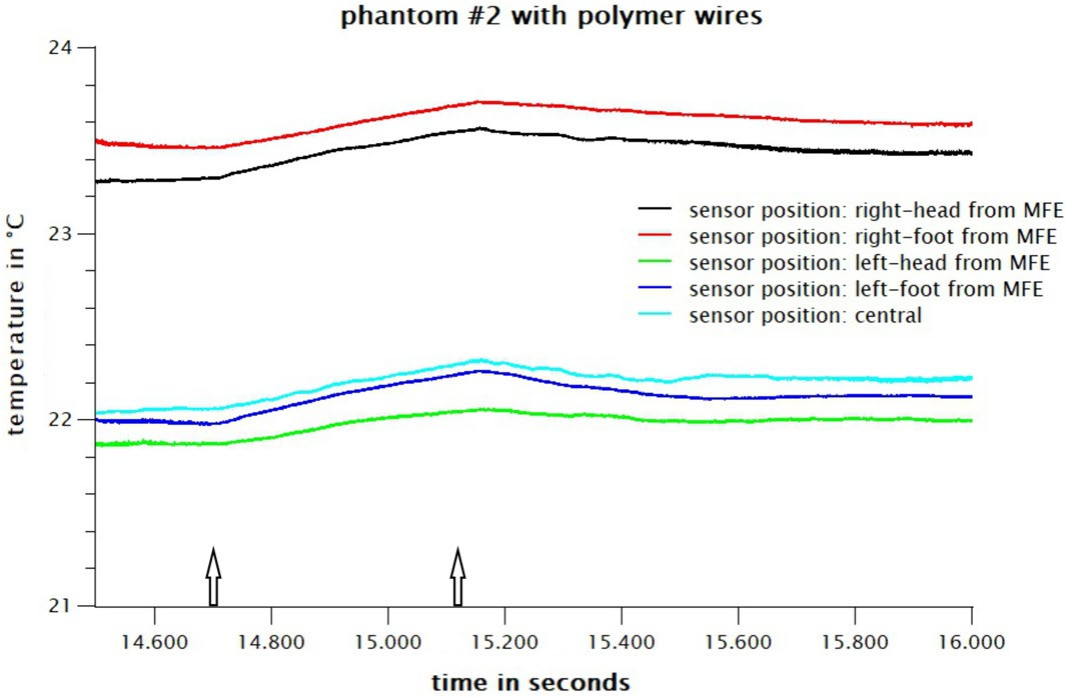
Heating effect of MRI on a polymer electrode with polymer cable loop. The central temperature sensors serves as control. No MRI induced heating can be observed. The image sequence starts at second 14.700 for 7 min and 26 s. The beginning and the end of the respective measuring sequence are marked with an arrow.

First, it is clearly evident that the electrodes do not interfere with imaging (Fig. 12). Only the temperature changes during the heating time are interesting, i.e. while the measurement sequence is running and RF is applied, i.e. between approx. 12220 s and 12640 s in Fig. 13 and 7 min from 14,700 in Fig. 14. The temperature sensors all have different offsets. For a better representation (otherwise all curves would overlap) we have not corrected them.

There are clear temperature peaks when using a copper cable of up to 3 °C in the immediate vicinity of the electrode and of about 1.5 °C at a distance of 5 cm (Fig. 13). When using the polymer cable, the heating drops well below 1 °C at all positions, which corresponds to the normal, technically conditioned general heating in the MRI (Fig. 14, image sequence starts at second 14.700). The central sensor position serves as control.

In summary, no significant heating can be measured with polymer electrodes and conductive textile wires during MRI. The replacement of the conductive textile wires with copper leads results in a steep rise of temperature during MRI.

The polymer electrodes show a stable impedance under incubator conditions with high humidity and temperature for 4 consecutive days.

## Discussion

So far, the PEDOT polymer electrode was used in the scientific context to record non-invasive EEGs in freely moving animals. The tight bond to the skin and the small size of the electrode, especially its flat appearance, minimizes relative movements between electrode and skin and hence notorious movement artifacts. But the electrode has additional advantages which are potentially useful for the clinical context: small size, completely metal-free, nearly invisible, no drying and pain-free removal even without water.

Long-term EEG recordings in the clinical context directly provoke the question, if the electrode is MR-safe. We were able to show that the temperature of the head phantom in the MRI-scanner is not rising when the PEDOT polymer electrode is used in combination with conductive textile cables (Fig. 14). Similar to the setup used here, the body coil was also used to test MRI-induced temperature changes in intracranial electrodes^20^ and the body coil was used to examine RF safety of EEG gel during MRI^21^. The imaging quality is not altered by the PEDOT polymer electrodes (Fig. 12). The electrodes do not cause disturbances caused by ferromagnetic or paramagnetic impurities. The bright areas under the electrodes are probably caused by the higher B1 field (the RF field used to excite the spin system) and do not disturb the image. On the contrary, one immediately has a marker for the actual position of the electrodes. It could also be that the electrode itself has absorbed some water, but that would not be a problem either.

These results suggest that the electrode could be an alternative to stiff carbon electrodes in the case of cardiac MRI and EEG fMRI. The extent to which the biosignal is disturbed by MRI needs to be clarified in future studies.

As expected, one gets a strong SAR increase in the area of the two electrodes in the simulation. If one leaves out the wire loop, then there is (as expected) no more substantial SAR overshoot, i.e. even with perfectly conducting electrodes (Fig. 11). The PEDOT polymer electrode in combination with the textile cable meets all conductivity criteria of the simulated sigma_20_electrode. In so far, the low heating effect of the polymer electrode in combination with the polymer cable in the magnetic field is strongly related to the relatively high impedance in comparison to a perfectly conducting (PEC) electrode in the simulation. In comparison to the heating, the good image quality in presence of polymer electrode and cable is most likely due to the absence of ferromagnetic influences, as mentioned earlier.

Even though the conductive textile wire was not insulated, it kept lower resistance in the climate chamber throughout the 4 days in comparison to the metal cable. Nevertheless, for future experiments we would insulate the wire for experiments in the climate chamber. Interestingly, the resistance values between the electrodes on the agarose plate remained constant even after one further day of drying outside the climate chamber after the 4 day experiment. This means that the results are not influenced by the air humidity. Unexpected was the relatively high impedance if we combined pure copper wire with the polymer electrode (Fig. 7). In the case of the resistance R we observed a steep rise at the second day (Fig. 6). Possible reasons are effects of air humidity or adverse interactions between PEDOT polymer electrode and the copper surface. Hence, we can not recommend the direct attachment of copper wire to the polymer electrode. Further measurements are necessary to understand the exact interaction between the PEDOT polymer electrode and metal cables, especially under high humidity conditions.

The PEDOT polymer electrode could be a timesaving solution for the neonatal intensive care unit, since it is stable under “incubator conditions” for several days and it must not be removed for MRI.

## Conclusion

Our results show, that the PEDOT polymer electrode in combination with the PEDOT textile cable is stable under conditions of high humidity and temperature and that heating of the electrode in strong magnetic fields (MRI) is below 1 °C, especially if it is used together with metal-free PEDOT textile cable.

## Supplementary Information

Supplementary Information.

## Data Availability

Some data are available in the supplementary material. Other data are available upon request at: ndecamp@jpberlin.de.
