# Supplementary material for: Physical behavior of PEDOT polymer electrode during magnetic resonance imaging and long-term test in the climate chamber
Source: Sci Rep. 2023 Apr 10;13:5826. doi: 10.1038/s41598-023-33180-5 (PMC10086067; doi:10.1038/s41598-023-33180-5)
Supplement: Supplementary file 1 — Supplementary Information. [file 41598_2023_33180_MOESM1_ESM.pdf]

DatenLogger

Konfiguration/Configuration

Benutzer/Operator	petesys UG
Intervall/Interval	00:05:00
Start möglich durch/Start possible by	Taste/Button
Startverzögerung/Start delay	00:00:00
Stopp möglich durch/Stop possible by	USB Verbindung/USB connect
Alarm/Alarm	Keine Alarme gesetzt/No Alarms set

Zusammenfassung/Summary

Startzeit/Start time	**27.11.2021 19:12:10**		Start durch/Start by	Taste/Button
Stoppzeit/Stop time	**01.12.2021 20:57:10**		Stopp durch/Stop by	USB Verbindung/USB connect
Datensätze/Records	**1174**		Dauer/Duration	**4 d 01:45:00**
	Min	Avg	Max	
Abs. Luftdruck/Abs. air pressure	**980.1 hPa**	**993.0 hPa**	**1002.6 hPa**	
Feuchtigkeit/Humidity	**45.2 %rH**	**84.0 %rH**	**91.7 %rH**	
Temperatur/Temperature	**25.1 °C**	**34.8 °C**	**35.8 °C**	
Taupunkt/Dew point	**17.3 °C**	**32.9 °C**	**35.3 °C**	

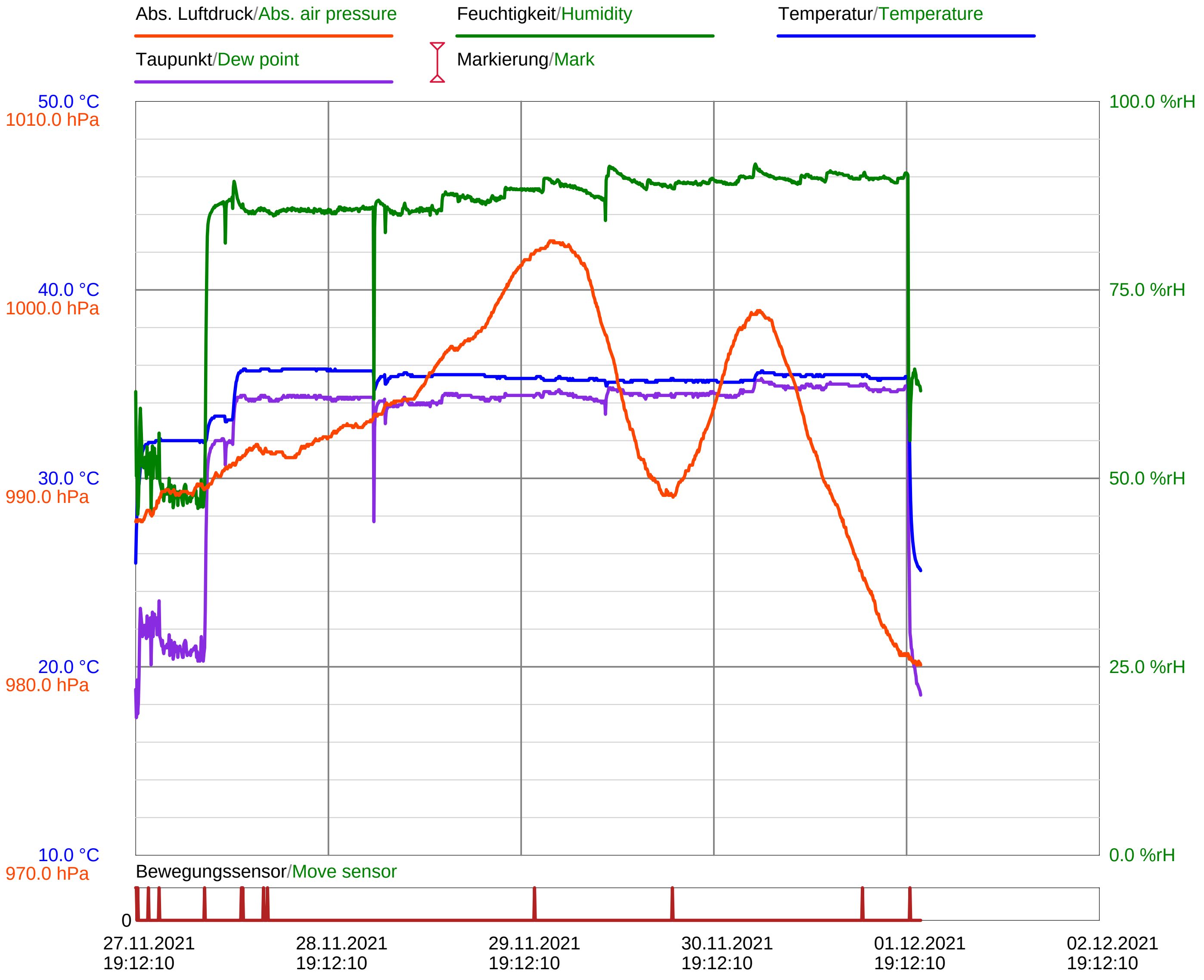

Unterschrift/Signature
